# Influenza viruses – antiviral therapy and resistance

**DOI:** 10.3205/id000030

**Published:** 2017-04-25

**Authors:** Susanne Duwe

**Affiliations:** 1 Robert Koch Institute, Division of Influenza Viruses and Other Respiratory Viruses, National Reference Centre for Influenza, Berlin, Germany

**Keywords:** infection, antiviral resistance, neuraminidase, surveillance, monitoring

## Abstract

Influenza is a serious and frequently underestimated, but vaccine preventable disease. The adamantane derivates rimantadine and amantadine and the neuraminidase inhibitors zanamivir and oseltamivir are the only antiviral drugs currently approved in Europe for therapy and prophylaxis of influenza infections. Resistance to these drugs occurs due to mutations within the therapeutic target proteins M2 ion channel protein and viral neuraminidase. An unexpected occurrence of oseltamivir-resistant seasonal A(H1N1) viruses was detected in winter 2007/2008. The prevalence of these viruses increased rapidly and nearby all viruses circulating during the following seasons were resistant to oseltamivir. The A(H1N1)pdm09 viruses replaced the former seasonal A(H1N1) subtype during the 2009–2010 influenza season. Fortunately, resistance to neuraminidase inhibitors was detected in A(H1N1)pdm09, A(H3N2) and influenza B viruses only sporadically and was treatment related mostly. Comprehensive analyses of circulating viruses showed a high prevalence of A(H3N2) influenza viruses that are resistant to adamantane derivates since 2004/2005 and a progressive trend in the prevalence of resistant viruses up to 100% in following seasons. The M2 ion channel protein of A(H1N1)pdm09 viruses is associated with the Eurasian avian-like swine lineage and thus show “natural” resistance to adamantane derivates. Therefore, only neuraminidase inhibitors are recommended for influenza treatment today.

This manuscript summarizes the occurrence and spread of antiviral resistant influenza viruses and highlights the importance for developing and/or approving new antiviral compounds.

## 1 Fundamental knowledge

Influenza viruses belong to the family *Orthomyxoviridae* that is characterized by the ability to attach on glycoproteins of host cell surfaces and a segmented genome composed of single stranded, negatively orientated ribonucleic acid (–ssRNA). Based on their molecular features and serological characteristics of their nucleoproteins and matrix proteins influenza viruses are divided into three genera: *Influenzavirus A*, *Influenzavirus B* and *Influenzavirus C* [1]. Whereas infections with influenza C viruses are often symptomless in humans, influenza A and B viruses cause annual epidemics known as seasonal flu, and influenza A viruses also cause pandemics at random intervals [2].

Influenza A viruses are zoonotic pathogens that can infect a broad range of species including birds, pigs and humans. According to the antigenic properties of their surface glycoproteins hemagglutinin (HA) and neuraminidase (NA) influenza A viruses are further divided into 18 HA and 11 NA subtypes (H1–H16 and N1–N9 in wild waterfowl, H17, H18 and N10, N11 in bats) [3]. In comparison to influenza A viruses influenza B viruses are less variable. They are antigenically related to either B/Victoria/2/87 or B/Yamagata/16/88 and are distinguished into two lineages which are referred to as the Yamagata and the Victoria lineage [4]. 

The life cycle of the influenza virus begins with binding of the virus particles to the surface of the host cells. Binding is mediated by the interaction of viral hemagglutinin (HA) with sialyloligosaccharides on proteins and lipids of the cell membranes. Due to receptor-mediated endocytosis the virus is internalized into the host cell enclosed by an endosome. Triggered by low pH in late endosomes and mediated by M2 ion channel, a conformational change of HA induces the fusion of the viral and the endosomal membrane. This triggers the release of uncoated viral ribonucleoprotein (vRNP) complexes into the cytosol of the host cell cytoplasm. After transport of vRNP complexes into the nucleus, replication and transcription follows the amplification of vRNA and synthesis of mRNAs for viral protein synthesis. Newly assembled vRNPs are exported to the cytoplasm and assembled with viral proteins at budding sites within the host cell membrane, followed by the budding and, after cleavage by neuraminidase, release of influenza virions [2].

Minor changes in viral proteins (antigenic-*drift*) are caused by high genomic variability of influenza viruses. Due to the lack of a proof-reading activity of the viral polymerase point mutations in the vRNA occur that might result in amino acid exchanges of the antigenic epitopes of the HA and NA proteins. This worsens the recognition and neutralization (abolishment of infectivity) of influenza viruses by the host’s immune system antibodies and thus may lead to immune evasive virus variants. Antigenic-drift is the cause for influenza epidemics by weakening the original bond between antigen and antibodies produced by vaccination, or infection induced immunity.

Antigenic shift results from a reassortment between at least two influenza subtypes resulting in a new influenza subtype. This may generate a novel virus able to infect the majority of the population due to their lack of immunity towards the unknown pathogen leading to high infection rates and pandemic spreads [3], [5]. Both mechanisms, point mutations as well as reassortment, can affect the efficacy of anti-influenza drugs. 

Influenza occurs globally with an estimated annual attack rate of 5–10% in adults and 20–30% in children. Both influenza A and influenza B viruses cause seasonal epidemics and out-of-season sporadic cases and outbreaks [6]. A typical influenza is marked by sudden onset of fever (≥38.5°C), dry cough, sore throat, body aches and pains, and headaches. Other symptoms may include general fatigue, sweating, rhinorrhea, but also nausea, vomiting, and diarrhea. In more severe cases, infection can lead to pneumonia, bacterial superinfection and even death. Nevertheless, not all patients fall ill with typical symptomatology. The duration of the disease is usually 5–7 days, which can be considerably longer depending on complications and risk factors [7]. 

Influenza is a vaccine preventable disease and influenza vaccines have been available for use in Europe since the 1960s. Currently most influenza vaccines contain three different influenza strains (trivalent): two influenza A strains (A(H1N1) and A(H3N2) subtypes) and one influenza B strain (Victoria or Yamagata lineage). Starting from the influenza season 2014–2015 new quadrivalent combination vaccines containing four different influenza strains are increasingly becoming available in the European Union/European Economic Area (EU/EEA). These vaccines contain two influenza A strains (A(H1N1) and A(H3N2) subtypes) and two influenza B strains (Victoria and Yamagata lineages) [8]. Annual vaccinations with the current antigen combination are recommended by WHO even when the antigen composition of the vaccine is unchanged compared with the previous season [6]. In Germany, the *Standing Committee on Vaccination* (STIKO) recommends annual vaccination in autumn as a standard vaccination for all persons aged 60 years and older, and where indicated in specific groups of persons e.g. children, adolescents and adults with an increased health risk resulting from an underlying disease, all pregnant women, persons at increased risk, e. g., medical personnel, persons in establishments dealing extensively with the public, as well as persons who may be possible sources of infection by caring for individuals at particular risk [9]. 

## 2 Currently available influenza medications

In Germany prescription medicines from two classes of active substances are approved for prevention and therapy of influenza infection (Table 1 (Tab. 1)). The M2 ion channel inhibitor amantadine belongs to the group of **adamantanes** and blocks the release of viral RNA into the cytoplasm of the host cell. This effect is achieved with therapeutic dosage of the active substance only with influenza A not with influenza B viruses because of different structure of the ion channel in both influenza species [10]. Due to the current resistance patterns of circulating viruses, the clinical use of adamantanes is not recommended presently (refer section 6). Thus, amantadine is currently not used for the treatment of influenza infections. 

The compounds **oseltamivir** (Tamiflu™) and **zanamivir** (Relenza™) belong to the group of neuraminidase inhibitors and were approved and authorized by the *European Medicines Agency* (EMA) for prevention and treatment of influenza in the European Union/European Economic Area (EU/EEA) in 2002 and 1999 [11], [12]. They inhibit selectively the neuraminidase of influenza A and B viruses. Thus, the release of new viruses from infected cells is prevented. 

**Oseltamivir** is admitted by EMA for adults and children including full-term newborns. It represents a prodrug and is available as oseltamivir phosphate in the form of capsules and as a powder for oral suspension. The compound is biotransformed by esterases in the intestinal tract and in the liver into the active metabolite oseltamivir carboxylate. The most common possible side effects include nausea, vomiting and headache. Antiviral therapy in neonates and young infants (<3 months) should be considered after careful risk assessment in severe clinical disease and should follow the recommendations of the *German Society of Pediatric Infectious Diseases* (DGPI) [11], [13].

**Zanamivir** showed unsatisfactory oral bioavailability and was therefore mixed with lactose and inhaled as a dry powder. Undesirable side effects are headache, diarrhea, nausea, and vomiting. Zanamivir is approved by EMA for treatment and prevention of influenza infections in patients from five years old. EMA issued in 2011 a summary on compassionate use for intravenous (i.v.) zanamivir in a specific targeted population and should be considered only to treat i.v. critically ill adults and children having a life-threatening condition [12], [14], [15]. 

In 2014 the neuraminidase inhibitors oseltamivir and zanamivir have been subject for critically discussion concerning their effectiveness and safety, as well as the appropriateness of stockpiling these drugs for use in future influenza pandemics [16], [17], [18], [19], [20]. An expert opinion from the *European Center for Disease Prevention and Control* (ECDC) and national authorities as well as national professional virological, medical and therapeutical associations (GfV e.V., DVV e.V, PEG e.V) confirmed earlier assessments that there is no significant new evidence from randomized control trials to support any changes to the approved indication and recommended use of neuraminidase inhibitors in EU/EEA Member States. This position was consistent with guidance from the *World Health Organization* (WHO) and other national public health organizations in the United States and Australia [21], [22], [23], [24]. 

**Peramivir** (USA: Rapivab™, Japan: Rapiacta™, South Korea: PeramiFlu™) is a cyclopentane neuraminidase inhibitor of influenza type A and type B viruses, which inhibits also various antiviral resistant influenza viruses. Due to its poor oral bioavailability peramivir was developed as intramuscular (i.m.) or intravenous (i.v.) applicable active substance. The most common adverse reaction is diarrhea [25], [26]. During the pandemic in 2009 peramivir was admitted in the USA based on an emergency use authorization (EUA) by the *U. S. Food and Drug Administration* (FDA) until June 23, 2010. On December 19, 2014 the FDA approved also peramivir for treatment of acute uncomplicated influenza in patients 18 years or older who have been symptomatic for more than two days. It is administered as a single i.v. dose [27]. In Japan and South Korea peramivir injection is approved since 2010 for the treatment of adults, children and infants with influenza [28].

**Laninamivir octanoate** (Japan: Inavir^®^) the octanoyl ester prodrug of laninamivir represents a zanamivir analogue. It is a long acting neuraminidase inhibitor (LANI) with therapeutic efficacy after one single nasal inhaled administration. Following inhalation laninamivir octanoate is absorbed by the epithelial cells lining the respiratory tract where it is rapidly hydrolyzed to the active laninamivir [29], [30], [31]. Laninamivir was approved for influenza treatment in 2010 and for prevention of influenza both in adults and children in 2013 in Japan [32].

**T-705** (Favipiravir, Avigan™) represents a prodrug for oral administration. It selectively inhibits a broad-sprectrum of RNA-dependent RNA polymerases and thus inhibits the viral gene replication of influenza viruses and nine other RNA virus families [33], [34]. The active metabolite, T-705 ribofuranosyltriphosphate (T-705RTP) is generated intracellularly via phosphorylation by various cellular kinases [35]. The incorporation of T-705RTP into viral RNA during replication leads to a high mutation rate that generates a nonviable viral phenotype (lethal mutagenesis) [36]. Since March 2014 favipiravir is only approved for influenza pandemic preparedness in Japan [37].

All currently approved drugs for influenza prophylaxis and therapy (ion channel blocker, neuraminidase and polymerase inhibitors) are so-called direct acting drugs (DAD). In contrast, **DAS181** (Fludase™), administered as an inhalable dry powder, is a broad spectrum host directed investigational influenza antiviral. It is being developed as a medication to prevent and treat infections due to common respiratory viruses like influenza, parainfluenza, and other viruses using cell surface sialic acids as receptors during attachment. Generated as a recombinant fusion protein composed of a sialidase catalytic domain derived from *Actinomyces viscosus* fused with a cell surface-anchoring sequence, it cleaves the linkages of sialic acid on host cells thereby removing the receptors for viral attachment [38]. This renders the cells inaccessible to infection by virus. It has also demonstrated activity in individual cases of parainfluenza in immunosuppressed patients [39]. The affect on viral load of influenza viruses and the safety and tolerability of the drug is currently being evaluated in phase 2 clinical trials in the USA [40].

## 3 Influenza antiviral resistance testing

Resistance to antiviral drugs is caused by subtype and inhibitor specific point mutations in the viral genes of the therapeutic target proteins. To date, no resistance was detected for the polymerase inhibitor T-705 until now. In contrast, ion channel blocker- and neuraminidase inhibitor-resistant influenza viruses with point mutations in gene segments coding for the M2 ion channel and neuraminidase, respectively, are known. These mutations can be detected by sequence-based technologies (classical sequencing, pyrosequencing) or, by the use of e.g. RT-PCR or melting point analysis of specific PCR amplification products (genotypic resistance analysis) [41], [42], [43], [44], [45]. These genotypic assays are advantageous as they can be performed with high sensitivity directly on the clinical specimen without the need for virus isolation. But, estimation of *in vitro* resistance to inhibitors on the basis of genotypic profiles is only reliable when the correlation between the presence of a specific point mutation and drug susceptibility of influenza viruses was proven. Such a mutation represents a molecular marker of resistance. In case of M2 ion channel blockers five molecular markers of resistance are known [46]. In contrast, in most cases estimation of *in vitro* resistance to neuraminidase inhibitors on the basis of genotypic profiles is unreliable, because currently insufficient amount of data are present to correlate between the presence of molecular marker of resistance and an actual in vitro resistance marked by an increase of the 50%-inhibitory concentration (IC_50_). Mutations causing the NA H275Y substitution (N1 numbering, subtypes A(H1N1)pdm09 and A(H5N1)) are the only ones consistently associated with increased levels of IC_50_-values to oseltamivir detected in NA inhibition assays (phenotypic resistance). 

The genotypic resistance analysis is considered the gold standard for M2 ion channel blockers, but phenotypic resistance analysis represents the gold standard for NAI (Table 2 (Tab. 2)) [47]. It measures the inhibition of viral neuraminidase activity and can be carried out by colorimetric, fluorometric or luminometric neuraminidase inhibition assays [48], [49]. Cell-based assays measuring the inhibition of viral replication are not recommended for NA inhibitor susceptibility testing, because they have been shown to lack sensitivity and reliability, and can give falsely high or low IC_50_ values [47], [50], [51]. All phenotypic methods require cultured virus but allow the determination of 50% inhibitory concentration, i.e. that concentration of active compound required to inhibit enzyme activity by 50%. The *WHO expert working group on surveillance of influenza antiviral susceptibility* (WHO antiviral working group, AVWG) has established a set of criteria to define the antiviral susceptibility of viruses based on the fold-change of their IC_50_ value compared to reference IC_50_ values. For influenza A viruses, use of normal (<10-fold increase), reduced (10–100-fold increase) and highly reduced (>100-fold increase) inhibition, and for influenza B viruses the same criteria but using <5-fold, 5–50-fold and >50-fold increases compared to the median for viruses from the same type/subtype/lineage showing ‘normal inhibition’ (NI), is recommended. A reduced susceptibility to NA inhibitors measured in phenotypic assays should be verified by the detection of the associated molecular resistance marker [52]. Generally, the clinical relevance of the resistance detected using the NA inhibition assay in combination with genotypic analysis needs to be further evaluated. The IC_50_ values should not be used to draw a direct correlation with the drug concentrations needed to inhibit virus replication in the infected human host, as clinical data to support such inferences are inadequate. Although, assessment of influenza virus susceptibility to NA inhibitors provides a reliable and reasonably comprehensive approach to the identification of NA inhibitor-resistant isolates for surveillance purposes, there is a pressing need to establish a clinically relevant IC_50_ cutoff value which could be used to differentiate statistical outliers from truly resistant viruses [52], [53]. 

## 4 Quality assurances of testing and monitoring antiviral susceptibility of influenza viruses

The implementation of genotypic and/or phenotypic tests for antiviral susceptibility is complex and the laboratory expertise required for validation and troubleshooting should be considered. Especially for neuraminidase inhibition assays, although some are commercially available (NA-Fluor, NA-Star, NA-XTD; Life Technologies), additional training is likely to be necessary [47], [49]. The use of genotypic methods also requires a good working knowledge of the encoded NA amino acid substitutions, known to confer reduced susceptibility to antivirals in specific influenza virus types and subtypes [47]. The standard operation protocols for in house NA inhibition assays provided from WHO addressed mainly the *National Influenza Centres* (NICs) but are also available for clinical laboratories interested in influenza antiviral resistance testing [54]. However, there is a strongly recommendation, that individual results of antiviral susceptibility testing in clinical laboratories should be collected nationally at the NIC [55].

The external quality assessment (EQA) exercise held for European influenza reference laboratories in 2011 has identified the need for harmonization antiviral susceptibility testing and data interpretation. Although interpretation of the data generally matched the consensus, only 85% of participants identified oseltamivir reduced susceptibility/resistance in a mixture of A(H1N1)pdm09 oseltamivir-sensitive/-resistant viruses and 23% considered oseltamivir-sensitive influenza B virus reduced susceptible/resistant [56]. The external quality assessment for influenza antiviral susceptibility for the *European Reference Laboratory Network for Human Influenza* (ERLI-Net) held two years later in 2013 showed that compared to 2010 the score improved, although in total only 85% of the oseltamivir results and for zanamivir 73% of results were correct [57]. 

When deciding whether implementation of antiviral susceptibility testing within a laboratory is practical together with the long-term costs and the requirements for testing, a training of laboratory staff in implementation and validation, troubleshooting, data analysis and interpretation should be considered. This is often best delivered through one-to-one training by a partner laboratory (referred to as “twinning”) [47], [56]. 

## 5 Monitoring of antiviral susceptibility of influenza viruses in Germany

Worldwide, except Europe, antiviral surveillance data were mainly reported via the *WHO Collaboration Centres* (WHOCC). In Europe antiviral resistance is monitored by the *European Centre for Disease Prevention and Control* (ECDC) and the *WHO/Europe Influenza Surveillance* (EuroFlu) based on the reports sent by influenza reference laboratories (NIC) to *The ECDC Surveillance System* (TESSy). In Germany, circulating influenza viruses were tested in the national influenza centre (NRZ Influenza; http://www.rki.de/DE/Content/Infekt/NRZ/Influenza/arbeitsbereiche/abeitsbereiche_node.html) for their antiviral susceptibility on a large scale by using genotypic and phenotypic methods, thus emergence and spread of resistant influenza viruses were monitored. The data were reported weekly to TESSy and additionally published via the weekly and seasonally reports of the *Working Group Influenza* (AGI; https://www.influenza.rki.de). The examination of a representative number of influenza A and influenza B viruses circulating in Germany between October 1998 and May 2016 yielded a resistance situation comparable with other countries worldwide. 

## 6 Resistance of influenza viruses to adamantane derivates

Resistance to the adamantane derivates amantadine and rimantadine can emerge rapidly during treatment. A prevalence of <1% was observed in circulating viruses until 2004. During the influenza season 2004–2005 a high prevalence and a strong spreading of community A(H3N2) isolates resistant to adamantanes were detected in Asia, Europe, Australia and the USA [58]. In the following winter 2005–2006 the prevalence of these viruses increased to nearly 100% [59]. These findings and the first reports on the detection of resistant seasonal A(H1N1) viruses rendered this class of drugs mostly ineffective [60]. In response, the CDC recommended against the use of adamantane derivates for treatment and prevention of influenza infections [61]. In Germany, the prevalence of primary resistance of A(H3N2) viruses to adamantane derivates was 12% during the 2004–2005 season. Resistance increased to 81% during the following winter 2005–2006. Very high frequency of resistant A(H3N2) viruses continued to be detected, with 100% in the 2007–2008 influenza season [62]. The resistance-associated S31N substitution (serine-to-asparagine) of the M2 ion channel protein (AGT→AAT) was the most commonly observed mutation [63]. In Germany, all tested A(H1N1) viruses remained sensitive to amantadin and rimantadin [62]. In the northern hemisphere increased prevalence of resistance among A(H1N1) viruses also has been observed, although the overall proportion of resistant A(H1N1) viruses was not as high as that for A(H3N2) viruses until 2009 [59]. The appearance of influenza viruses of subtype A(H1N1)pdm09 led to a pandemic in 2009, followed by complete A(H1N1) subtype replacement. Due to reassortment (antigenic shift) the A(H1N1)pdm09 viruses carry the M2 ion channel protein originated from the Eurasian avian-like swine lineage [64]. Viruses that are associated with this lineage possess the M2-S31N substitution as a polymorphism, and show thus “natural” resistance to adamantane derivates [65], [66].

## 7 Resistance of influenza viruses to neuraminidase inhibitors

Resistance to the neuraminidase inhibitors oseltamivir and zanamivir following EMA and FDA approval occurred only sporadically and mostly on clinical trials in immunocompromised patients. The incidence of oseltamivir resistant influenza A(H3N2) and A(H1N1) viruses seen in clinical trial samples of immunocompetent patients until July 2004 was 0.33% (4/1228) in adults (≥13 years) and 4% (17/421) in children (≤12 years), resulting in an overall incidence of 1.26% [67], [68]. An high degree of A(H1N1) and A(H3N2) oseltamivir-resistant viruses has been found within two clinical trials [67], [69]. During oseltamivir treatment about 16.3% (7/43) resistant A(H1N1) and 18% (9/59) resistant A(H3N2) viruses occurred, which was may be connected to an insufficient dosage regime. 

In contrast to these clinical findings, the occurrence and spread of neuraminidase inhibitor-resistant strains within seasonal influenza viruses was a rare event until winter 2007. In comparison to sensitive viruses the resistant viruses showed a diminished viral fitness, marked by worse replication rates, and lower transmissibility, leading to the conclusion that resistance-associated mutations are unlikely to be of clinical consequence [46], [70], [71]. Therefore, the detection and rapid spread of oseltamivir-resistant A/Brisbane/59/2007-like A(H1N1) viruses in November 2007 within the seasonally circulating influenza viruses was unpredicted and unexpected [72]. All resistant viruses carried the H275Y (histidine-to-tyrosine) substitution in their neuraminidase and showed highly reduced susceptibility to oseltamivir. During the following season 2007–2008 these viruses spread rapidly over the northern hemisphere. The resistance to oseltamivir could not be explained with reassortment and was not the consequence of widespread therapeutically usage of oseltamivir [73], [74]. The infected patients were not linked epidemiologically and showed symptoms that were similar to that of infections with sensitive viruses [75], [76]. The oseltamivir-resistant viruses were shed by adults up to eight days and were transmissible by household contacts without any change in their resistance profile [76]. They did not show obvious attenuation relative to earlier viruses carrying the H275Y substitution [77]. During the summer 2008 the prevalence of oseltamivir-resistant A(H1N1) viruses increased up to 80% within the southern hemisphere. In the following season 2008–2009 almost all circulating A(H1N1) viruses were oseltamivir-resistant [78], [79]. Bloom et al. showed that two permissive secondary mutations (V234M, R222Q) occurred in seasonal A(H1N1) shortly before the widespread appearance of H275Y. These mutations restore the detrimental influence of the NA-H275Y mutation and thus enable the evolution of influenza oseltamivir resistance [80].

In 2009, a triple reassortant swine virus reassorted with an Eurasian avian-like swine virus resulting in the A(H1N1)pdm09 virus, that has spread internationally with unprecedented speed and caused the first pandemic of the 21^st^ century [64], [78], [81]. The A(H1N1)pdm09 neuraminidase originated from the Eurasian avian-like swine A(H1N1) virus and is characterized by neuraminidase inhibitor sensitivity [64]. The former oseltamivir-resistant seasonal A(H1N1) virus become to extinct soon after the emergence of the A(H1N1)pdm09 virus [78].

In Germany, overall 3,720 influenza viruses (66% A(H1N1)pdm09, 19% A(H3N2) and 15% influenza B) circulating between April 2009 and May 2016 were analyzed phenotypically and/or genotypically [82]. Only twelve out of 2,430 A(H1N1)pdm09 viruses showed highly reduced susceptibility to the neuraminidase inhibitor oseltamivir indicated by an up to 1,000-fold increase of the IC_50_ for oseltamivir in phenotypic assays compared to the sensitive wild-type virus. All these resistant viruses carried the substitution H275Y in their neuraminidase and remained sensitive to zanamivir. Almost all viruses were collected from patients during or after oseltamivir treatment. Only, two pandemic viruses showed resistance to oseltamivir despite the lack of any evidence that they arose during antiviral treatment. In the first case, the virus was isolated from a respiratory sample of a 21-year-old man who developed an influenza-like illness while staying abroad for vacation. The second case was a 9-year-old otherwise healthy boy. The oseltamivir-resistance was confirmed by phenotypic analysis of the virus isolates. Pyrosequencing analysis performed on the patients’ respiratory specimens showed the molecular resistance marker NA-H275Y. In both cases the viruses remained sensitive to zanamivir in phenotypic assays with IC_50_<2nM and further molecular markers of resistance in viral NAs were not detected [42], [62], [83]. A(H3N2) viruses with altered susceptibility to neuraminidase inhibitors were detected in two cases. In 2012 treatment-selected A(H3N2) viruses showing reduced susceptibility to neuraminidase inhibitors due to the NA-R292K substitution were isolated from two children at day five of oseltamivir treatment [84]. Influenza B viruses showing reduced susceptibility to neuraminidase inhibitors were not detected. 

Globally, about 99% of the tested influenza viruses were susceptible against the four neuraminidase inhibitors. The prevalence of viruses with reduced or highly reduced sensitivity to neuraminidase inhibitors was highest with 1.9% in the 2013–2014 influenza season, compared to the 2012–2013 and 2014–2015 seasons where the frequencies were 0.6% and 0.5%, respectively [85]. The increase of resistant viruses in 2013–2014 is associated with the detection of several clusters of A(H1N1)pdm09 viruses carrying the H275Y substitution in China, Japan and the United States [86]. In A(H3N2) and influenza B viruses, mutations, known to confer reduced susceptibility to neuraminidase inhibitors or phenotypically reduced sensitivity are being detected at a substantially lower frequency than in A(H1N1)pdm09 viruses [78].

## 8 Conclusions

Influenza virus infection is a serious and frequently underestimated disease. Due to the development of adamantane resistance in A(H3N2) and the emergence of adamantane-resistant A(H1N1)pdm09 viruses in 2009, the neuraminidase inhibitors zanamivir and oseltamivir are the only effective antiviral drugs, available commercially in Europe. They act against all of the currently circulating human influenza viruses. Although reduced susceptibility to these antivirals remains rare, the appearance and spread of resistant viruses in the past underlines the urgent need to develop and/or approve new active compounds and to closely monitor the resistance profiles of circulating influenza viruses in the international context.

## Notes

### Acknowledgement

This review was supported by the Scientific Advisory Board for Antiviral Therapy of the German Association for the Control of Virus Diseases (DVV) and the Society of Virology (GfV) as well as the Section for Antiviral Therapy of the Paul-Ehrlich-Gesellschaft (PEG). The author's grateful thanks are given to PD Dr. Michaela Schmidtke for critically reviewing and discussing the manuscript. 

### Competing interests

The author declares that she has no competing interests.

## Figures and Tables

**Table 1:**
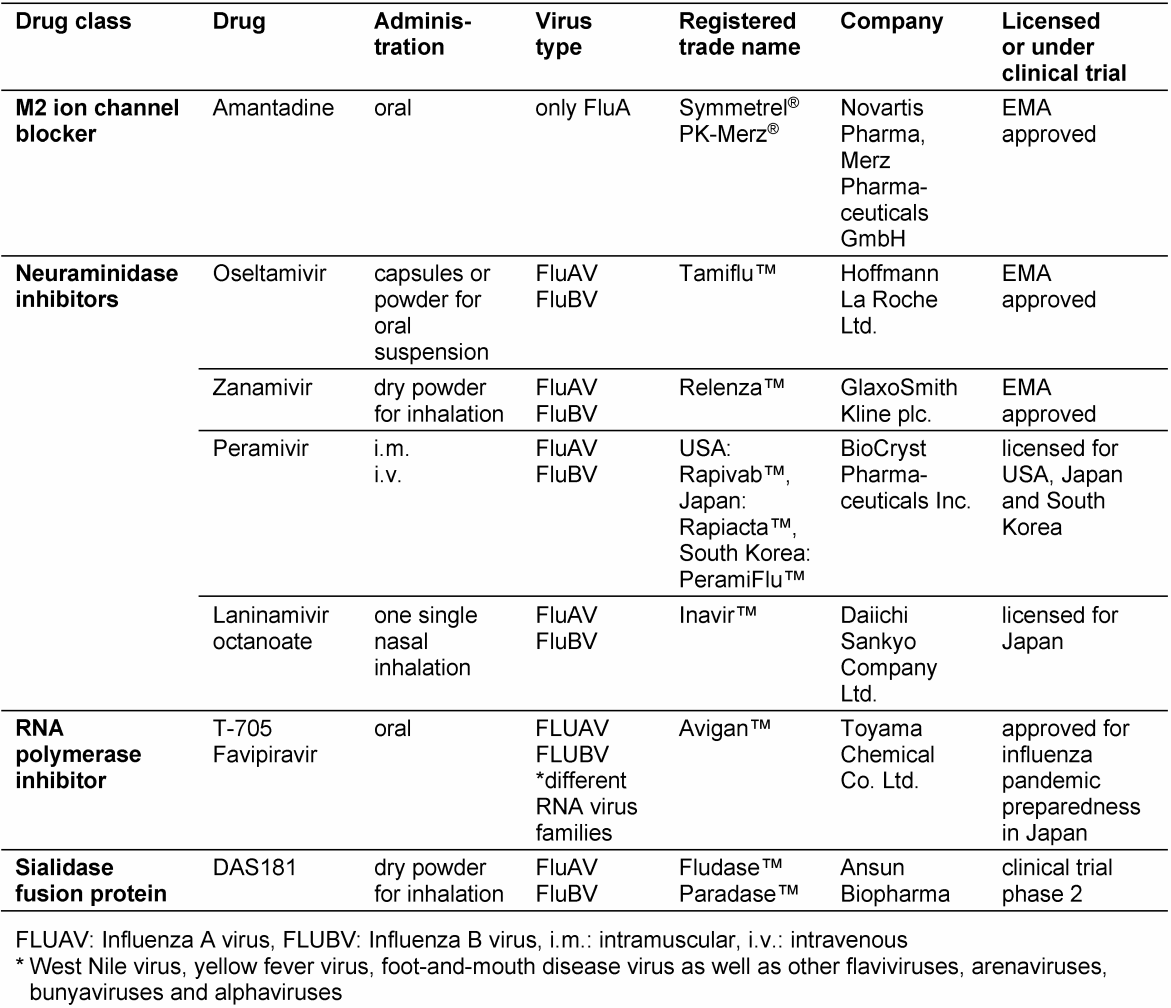
List of anti-influenza drugs recently approved or under clinical trials

**Table 2:**
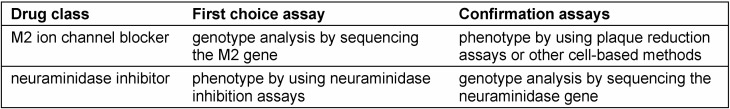
Influenza antiviral resistance testing
